# Novel Two-Infusion Pump Technique for Exchange Transfusion in a Hyperbilirubinemic Neonate

**DOI:** 10.7759/cureus.54012

**Published:** 2024-02-11

**Authors:** Naramreddy Sudheesh Reddy, Aditi Rawat, Sagar Karotkar, Ashish Varma, Amar Taksande, Revat J Meshram, Chaitanya Kumar Javvaji, SreeHarsha Damam

**Affiliations:** 1 Pediatrics, Jawaharlal Nehru Medical College, Datta Meghe Institute of Higher Education and Research, Wardha, IND; 2 Neonatalogy, Jawaharlal Nehru Medical College, Datta Meghe Institute of Higher Education and Research, Wardha, IND

**Keywords:** novel approach, infusion pumps, exchange transfusion, abo incompatibility, neonatal hyperbilirubinemia

## Abstract

Neonatal hyperbilirubinemia is a common concern in newborns, with ABO blood group incompatibility serving as a significant risk factor for severe jaundice. This case report outlines the successful management of a 2.5 kg female infant born to a primigravida mother with ABO incompatibility-induced hyperbilirubinemia. The neonate, born at 38.4 weeks via lower segment cesarean section, exhibited signs of jaundice at 91 hours of life, prompting screening and subsequent confirmation of serum bilirubin levels 26.4. The decision was made using the American Academy of Pediatrics (AAP) and categorized the child under high risk according to age and bilirubin level to implement a complete exchange transfusion using a novel approach with two infusion pumps. The unique aspect of this case lies in introducing a two-infusion pump technique, one to infuse and one to extract blood by inserting the IV set in opposite directions in the infusion pump to perform the exchange transfusion, aiming to minimize complications associated with traditional methods. Careful handling of umbilical venous and arterial lines, coupled with aseptic precautions, sought to mitigate the risk of sepsis. The procedure, conducted over two hours, demonstrated stability in vital signs and was monitored with a transcutaneous bilirubinometer. Post-transfusion, repeat serum bilirubin tests showed a decrease in bilirubin of 10.1, indicating the success of the novel exchange transfusion method. The infant was discharged after a five-day hospital stay, showcasing this innovative approach's potential efficacy and safety. This case contributes to the evolving strategies in neonatal care and emphasizes the importance of tailored interventions in managing hyperbilirubinemia associated with ABO incompatibility.

## Introduction

Hyperbilirubinemia, which is the most common clinical problem encountered in the neonatal period of infants, is an important neonatal problem that may have potentially toxic effects on the central nervous system such as kernicterus, seizures, and permanent neurodevelopmental damage in newborns and increases the stress burden on children [1]. Among the various etiologies, ABO blood group incompatibility has been identified as a significant risk factor for severe hyperbilirubinemia in newborns [2]. ABO incompatibility is a condition that occurs when a mother's blood type is incompatible with her baby's blood type, which can lead to hemolytic disease of the newborn (HDN). The prevalence of ABO incompatibility varies depending on the ethnic group and location. In general, ABO hemolytic disease is seen in 0.3-0.8% of Caucasian pregnancies, but it is more severe and more frequent at 3-5% in Asian or African pregnancies [3].

The distribution of blood groups in a compatible population is typically 45% for blood group O, 30% for blood group A, 20% for blood group B, and 5% for blood group AB [4]. The incidence of ABO HDN is about 2% of all births, but severe hemolytic disease occurs in only 0.03% of births [5]. Risk factors for ABO incompatibility are present in 12-15% of pregnancies, but evidence of fetal sensitization (positive direct Coombs test) occurs in only 3-4% [6]. First-born infants have a 40-50% risk for symptomatic disease, and progressive severity of the hemolytic process in succeeding pregnancies is a rare phenomenon [6-8].

The management of severe hyperbilirubinemia often necessitates exchange transfusion that is decided according to the baby's birth week, weight, and accompanying health problems. The procedure aimed at rapidly reducing serum bilirubin levels [2]. Traditional methods of exchange transfusion, where blood is extracted using the gravity method, have been associated with complications, including sepsis and hemodynamic instability [9]. In response to these challenges, our case introduces a novel technique employing two infusion pumps for the exchange transfusion, focusing on minimizing complications while ensuring optimal clinical outcomes. The present case report addresses the successful management of a neonate born with ABO incompatibility-induced hyperbilirubinemia through a pioneering approach to exchange transfusion.

## Case presentation

A female infant weighing 2.5 kg was born to a primigravida mother at 38.4 weeks of gestational age through a lower segment cesarean section due to oligohydramnios, with an amniotic fluid index of three, in a private nursing home. According to the mother's history, the infant cried immediately after birth and was placed by her side, and breastfeeding was given. However, at 91 hours of life, the infant displayed reduced activity and appeared jaundiced. A transcutaneous bilirubinometer revealed a value exceeding 20. Subsequent total serum bilirubin testing confirmed a level of 26.4, where conjugated bilirubin was 0 and unconjugated bilirubin level was 26.4, warranting an exchange transfusion.

**Figure 1:**
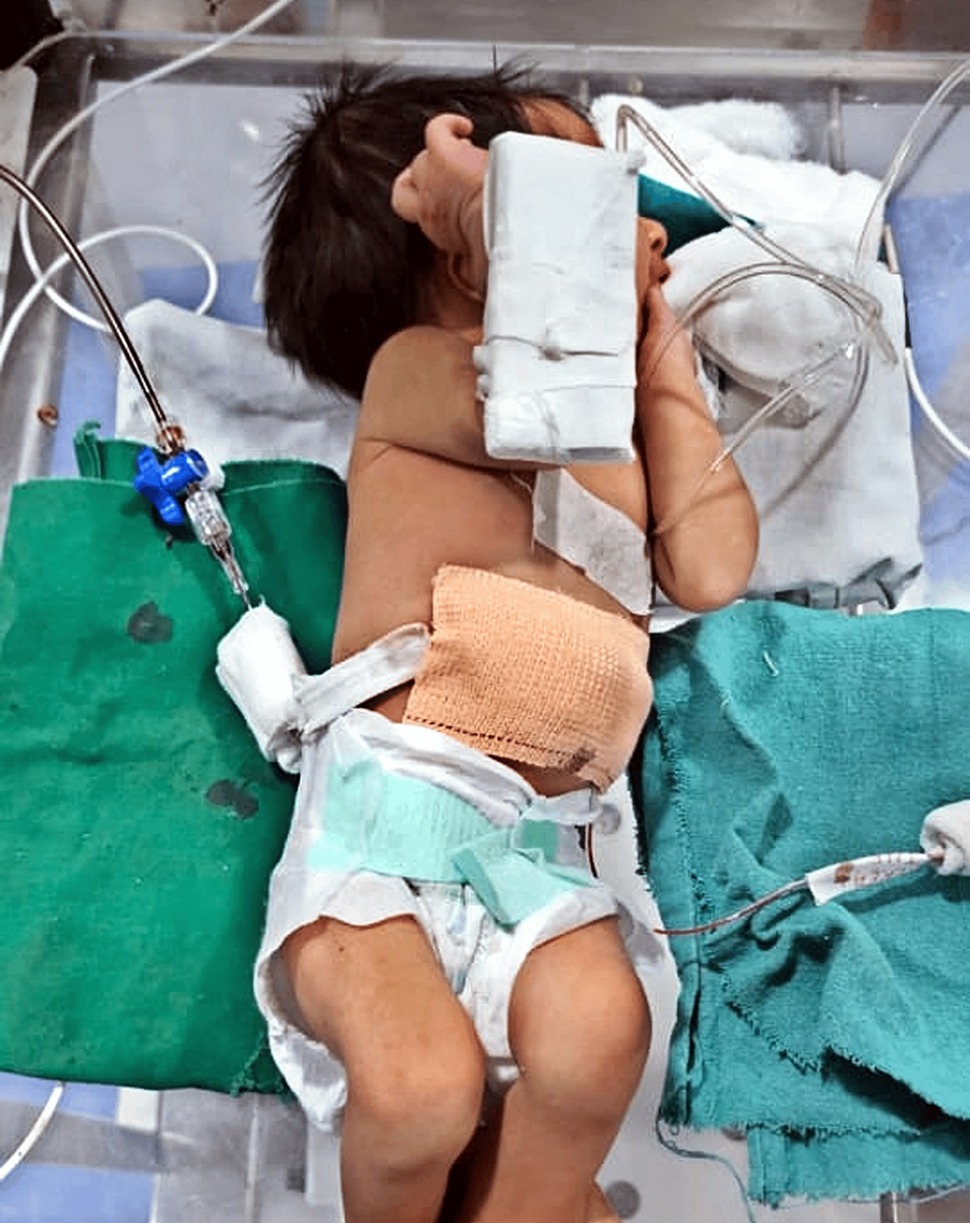
The on-admission photo of the infant in the intensive care unit

The infant was then referred to a tertiary care multispecialty hospital for further management. Upon receiving the infant, a thorough history and physical examination were conducted before transferring the infant to the neonatal intensive care unit (Figure 1).

Initial blood tests, including liver function tests, indicated a total serum bilirubin of 26.4, with unconjugated bilirubin accounting for the entire amount of 26.4. Hence, the Coombs test was done that showed positive results, whereas the blood test showed a decrease in reticulocyte count 7 (ref: 20000-80000 cells/uL) and grouping revealed the infant to be O positive, in contrast to the A positive blood type of the mother, suggesting ABO incompatibility.

Subsequently, the pediatrician and the medical team decided on a complete exchange transfusion, other laboratory test results were normal, and the infant was vitally stable and was on formula feed. The parents were briefed about the infant's condition, and written consent for the procedure was obtained. This marked the first instance in the hospital where a complete exchange transfusion was planned, utilizing two infusion pumps. This novel approach avoided complications associated with traditional pull and push techniques, such as sepsis and hemodynamic instability.

**Figure 2:**
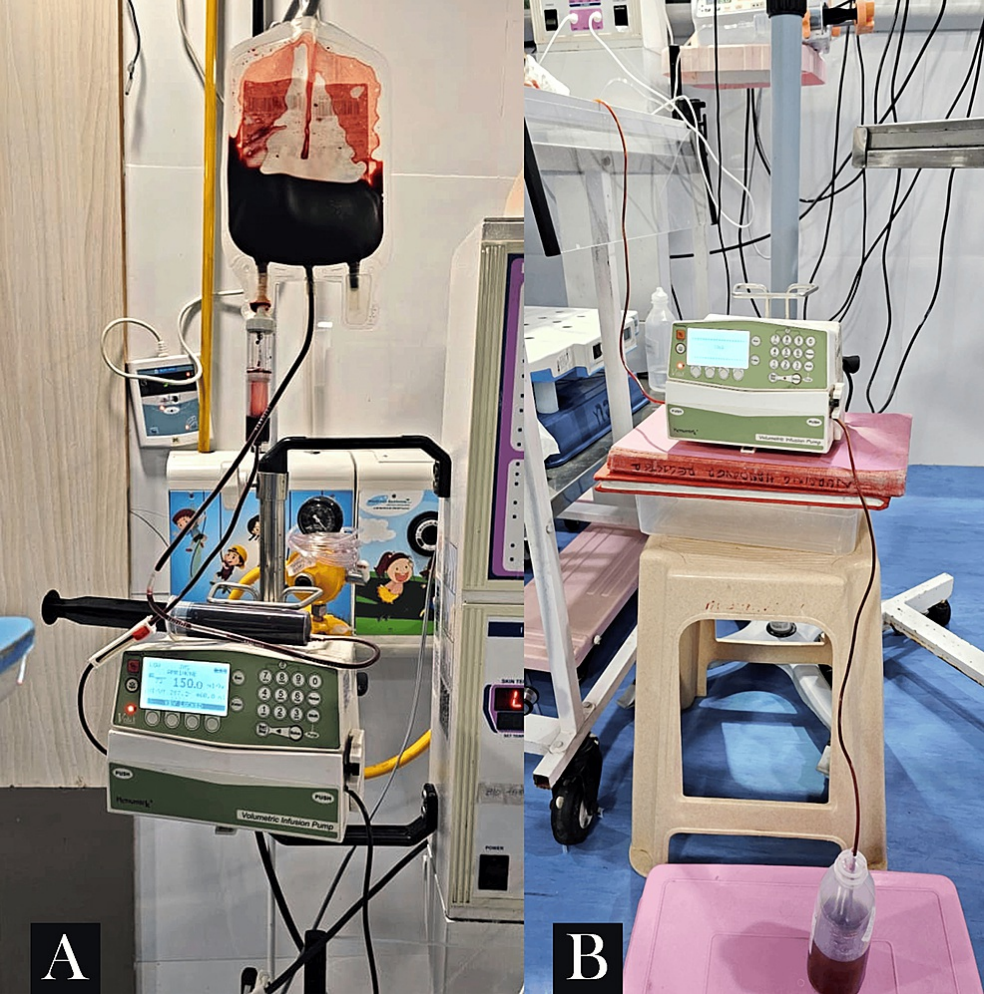
(A) Shows that the infusion pump was attached to the blood bag connected to the umbilical venous cannula, infusing blood at 150 mL/hr over one hour. (B) Shows other end was connected to an underwater seal, as depicted

O-negative blood was arranged for the exchange transfusion, with the calculated volume to be transfused and removed set at 150 mL over two hours. Two lines, an umbilical venous cannula and an umbilical arterial cannula, were secured for the procedure. One infusion pump was attached to the blood bag connected to the umbilical venous cannula, infusing blood at 150 mL/hr over one hour (Figure 2A). The challenge arose during the removal of blood from the infant, utilizing the umbilical arterial catheter connected to the second infusion pump set at a rate of 150 mL/hr. The other end was connected to an underwater seal, as depicted in Figure 2B. The arterial blood flow was in the direction of gravity.

Both blood flow rates were maintained at 150 mL/hr without interruption for two hours, with stable vitals recorded throughout the procedure, heart rate of 136 bpm, SpO_2_ of 96%, well-felt peripheral pulses, and a capillary refill time of less than three seconds. The procedure minimized handling the infant and the lines, reducing the risk of sepsis compared to conventional methods. Metabolic and vital parameter derangements were unlikely with this approach. Post-procedure, a repeat serum bilirubin test after six hours showed a decrease, indicating the success of the new method with minimal risk of complications. Repeat samples after the procedure further confirmed the reduction in serum bilirubin levels, accompanied by clinical improvement. The infant was discharged after a five-day hospital stay.

## Discussion

The presented case highlights the successful application of a novel approach to exchange transfusion in a neonate with ABO incompatibility-induced hyperbilirubinemia. A careful review of the literature underscores the significance of this case in optimizing the management of neonatal jaundice, especially in cases where conventional methods may pose heightened risks. The choice of ABO incompatibility as the underlying cause of hyperbilirubinemia in this infant is consistent with previous studies that have identified ABO blood group incompatibility as a significant risk factor for neonatal hyperbilirubinemia [10]. In this case, the successful outcome emphasizes the importance of recognizing and addressing this risk early in the neonatal period.

The decision to perform a complete exchange transfusion using two infusion pumps differs from traditional pull and push techniques. While exchange transfusions are a well-established method for severe hyperbilirubinemia, the novel use of two infusion pumps merits discussion. The rationale behind this approach is grounded in the desire to minimize complications associated with conventional methods, such as sepsis and hemodynamic instability [11]. The successful application of this technique in the present case supports the notion that innovation in procedural methods can contribute to improved patient outcomes. A key consideration in the presented case is the meticulous handling of the umbilical venous and arterial lines, undertaken with aseptic precautions. This approach aimed to reduce the risk of sepsis, a common complication associated with exchange transfusions [12]. We are incorporating two infusion pumps in the procedure to allow for a steady and controlled blood exchange, contributing to the overall stability of vital parameters during the transfusion.

The stability of vital signs throughout the exchange transfusion, as demonstrated by a heart rate of 136 bpm, SpO_2_ of 96%, well-felt peripheral pulses, and a capillary refill time of less than three seconds, further validates the safety and efficacy of the employed technique. Maintaining stable vitals is crucial, as fluctuations during the procedure can lead to adverse outcomes [13]. The successful reduction in serum bilirubin levels post-procedure, along with clinical improvement, underscores the effectiveness of the two-infusion pump approach. This outcome aligns with the overarching goal of exchange transfusions, which is to rapidly reduce bilirubin levels to prevent the neurotoxic effects of hyperbilirubinemia in neonates [14].

## Conclusions

In conclusion, the presented case underscores the successful implementation of a novel approach to exchange transfusion in a neonate with ABO incompatibility-induced hyperbilirubinemia. Using two infusion pumps during the procedure, aimed at minimizing complications associated with conventional methods, demonstrated both safety and efficacy. The meticulous handling of umbilical venous and arterial lines with aseptic precautions reduced sepsis risk. While further studies are needed to validate the broader applicability and safety of this approach, the present case offers valuable insights into optimizing the management of neonatal jaundice, particularly in cases involving ABO incompatibility.
